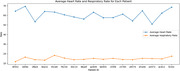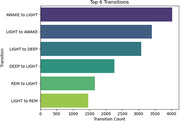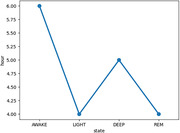# Exploring Sleeping Patterns of Patients with Dementia Based on Different Sleep States

**DOI:** 10.1002/alz.087978

**Published:** 2025-01-09

**Authors:** Dalene Ephme Ben George

**Affiliations:** ^1^ Sri Sivasubramaniya Nadar College Of Engineering, Chennai, Tamil Nadu India

## Abstract

**Background:**

Sleep patterns and disruptions may associate with increased dementia risk and contribute to its progression and cognitive decline. Understanding the complexity of the sleep‐dementia relationship is crucial for developing interventions that may delay cognitive decline and enhance the well‐being of individuals with dementia. This study seeks to explore how the sleeping patterns of patients with dementia impact them and aims to provide insights to help improve the sleep patterns of individuals affected by dementia.

**Method:**

The study uses the sleep data from TIHM project (Surrey and Borders Partnership, NHS Foundation), encompassing records of 17 dementia patients. The dataset contains details including sleep states, heart rate, respiratory rate, and snoring. Firstly, the average heart rate and respiratory rate are analyzed over time for each patient. Sleep transition states are explored to show patterns of transitions between different sleep states. The study explores circadian aspects by calculating average hours for each sleep state, and it provides additional insights, including average heart rate and respiratory rate based on sleep state and snoring.

**Result:**

This study provided significant insights into the sleep patterns of individuals living with dementia. The heart rates of patients vary from 50.87 to 69.43 with an average 60.57, slightly elevated from the normal. The respiratory rates range from 11.80 to 18.34, with an average 14.83 (Figure 1). Notably, individuals who snore exhibit a lower average heart rate (56.4) compared to non‐snorers (61.03). Sleep state analysis indicates slightly higher heart and respiratory rates during Awake and Rem states compared to other states. Sleep state transitions highlight a predominant shift from Awake to Light, followed by Light to Awake (Figure 2). Patients primarily spend time in the Awake state (6 hours), followed by Deep (5 hours), and then Light and Rem states (4 hours) (Figure 3).

**Conclusion:**

The study provides significant insights about the relationship between sleep patterns and dementia. Elevated heart and respiratory rates during Awake and Rem states underscore physiological aspects in individuals with dementia, with prevalent transitions from Awake to Light in sleep state analysis. These findings contribute to a deeper understanding of sleep in dementia, suggesting implications for enhanced care.